# Morphological Changes and MRI Characteristics of the Achilles Tendon in Amateur Marathon Runners With Different Running Experience

**DOI:** 10.1002/jfa2.70125

**Published:** 2026-01-11

**Authors:** Wanzhen Yao, Yong Chen, Siyu Dai, Jing Zhou, Xinmiao Mao, Yanjing Zhang, Jianping Ding, Jie Liu, Jie Huang

**Affiliations:** ^1^ Department of Radiology Hangzhou Normal University Affiliated Hospital Hangzhou Zhejiang China; ^2^ Hangzhou Institute of Sports Medicine for Marathon Hangzhou Zhejiang China; ^3^ Faculty of Medicine The Chinese University of Hong Kong Hong Kong China; ^4^ School of Clinical Medicine Hangzhou Normal University Hangzhou China

**Keywords:** Achilles tendon, marathon, morphological changes, MRI, running experience

## Abstract

**Background:**

With the escalating popularity of marathon running, Achilles tendon injuries, particularly gradual‐onset Achilles tendon injury, have become common, often causing substantial training disruptions. However, the influence of running experience on the Achilles tendon structure in amateur runners remains largely unclear. This study aimed to investigate the association between running experience and asymptomatic Achilles tendon pathology as well as its structural changes.

**Methods:**

This was a cross‐sectional observational study. Forty‐eight amateur marathon runners were categorized into four groups based on running experience (1, 3, 5, and > 5 years), with 12 healthy nonrunners as controls. Inclusion and exclusion criteria were strictly applied. All participants underwent MRI scanning using a 3.0 T GE scanner. Two radiologists evaluated MRI scans for pathology and measured tendon length, thickness, volume, and cross‐sectional area (CSA). Statistical analyses, including Shapiro–Wilk, ANOVA, Kruskal–Wallis H, and chi‐squared tests, were conducted using SPSS 23.0.

**Results:**

Baseline characteristics showed no significant group differences. Qualitative analysis revealed that the prevalence of midportion tendinopathy, insertional tendinopathy, and retrocalcaneal bursitis increased significantly with longer running experience. Quantitative measurements indicated that tendon thickness, volume, and CSA were significantly greater in long‐running groups compared to short‐running and control groups, whereas tendon length remained unchanged. Interobserver reliability was excellent.

**Conclusion:**

In amateur marathon runners, running experience is associated with increased asymptomatic Achilles tendon pathology and morphological remodeling. Prolonged running may induce both adaptive and degenerative changes, highlighting the importance of MRI‐based monitoring for early intervention in high‐risk populations.

## 1 Background

Marathon running has witnessed a remarkable global proliferation, with amateur runners accounting for the vast majority of participants in major events [1, 2]. This surge underscores the need to characterize the cumulative mechanical impact on the musculoskeletal system, particularly the Achilles tendon, which withstands repetitive loads of up to 6∼12 times body weight during propulsion [3, 4]. Epidemiological data from Ljubljana Marathon participants reveal a 53% lifetime prevalence of running‐related injuries, with the Achilles tendon and ankle ranking among the most affected sites [5]. Despite this, the relationship between running experience and subclinical structural changes in asymptomatic amateurs remains poorly defined, particularly regarding how cumulative mileage influences tendon morphology over years.

Cross‐sectional imaging studies have uncovered a high burden of MRI‐detectable abnormalities in asymptomatic runners [6]. Lohman et al. [7] identified bone marrow edema‐like signals in 19.1% of asymptomatic individuals, primarily in the calcaneus and talus, with lesions correlating significantly with running intensity. In a subsequent study, Yao et al. [6] discovered through MRI analysis of the ankle joints of 113 asymptomatic amateur marathon runners that 49 individuals (26.8%) exhibited Achilles tendinopathy and 21 individuals (11.5%) had Achilles tendon effusion. These findings align with observations from ultramarathon runners, where Freund et al. [8] documented a 14.7% increase in Achilles tendon diameter and widespread bone marrow edema after a 4487 km race, suggesting a continuum of adaptive to degenerative changes with prolonged loading.

Disorders of Achilles tendon can be divided into ruptures and overuse injuries. The latter include midportion and insertional tendinopathy, paratendinopathy, and superficial and retrocalcaneal bursitis [9]. Tendon remodeling is a dynamic process primarily influenced by mechanical loads, with running serving as a crucial regulator of this adaptive response. Analyses of risk factors underscore the relationship between running history and tendon health [4, 10]. The study reveals that runners have a larger Achilles tendon CSA and greater Achilles tendon stiffness compared to nonrunners [11, 12]. This finding suggests that the tendon adapts to accommodate the increased mechanical demands associated with running. Additionally, another cross‐sectional study found a high prevalence of tendon pathology among asymptomatic male distance runners. Notably, a correlation exists between the number of years spent running and the presence of pathology in the Achilles tendon [13]. This observation underscores the cumulative impact of mechanical loading over time, which may predispose tendons to structural disorganization, including collagen fiber misalignment and increased CSA. Although tendon hypertrophy, characterized by an increased CSA, is often an adaptive response to loading, it may also indicate underlying degeneration.

Despite these insights, critical knowledge gaps persist. Participation in a marathon can trigger a temporary thickening of the Achilles tendon as an initial adaptive response to sudden mechanical stress. However, it is unclear whether this transient thickening may progress into a chronic collagen disorder with continued running. A chronic collagen disorder is characterized by persistent degradation and disorganized alignment of collagen fibers within the tendons, potentially resulting in functional limitations and an elevated risk of injuries. The morphological and pathological changes of the Achilles tendon in amateur runners with varying levels of running experience have not been systematically assessed.

This study addresses these limitations by systematically analyzing Achilles tendon morphology and MRI characteristics in asymptomatic amateur runners stratified by running experience (1, 3, 5, > 5 years). Using high‐resolution MRI, we aim to (1) quantify the prevalence of pathologies (e.g., tendinopathy and retrocalcaneal bursitis) across experience levels; (2) assess longitudinal changes in tendon thickness, volume, and cross‐sectional area; and (3) determine how the structure and pathology of the Achilles tendon are influenced by increased running experience. By bridging acute and chronic perspectives, this research seeks to establish evidence‐based benchmarks for MRI screening in recreational athletes and inform targeted interventions to reduce overuse injury risk.

## 2 Methods

### 2.1 Study Design and Participant Recruitment

This cross‐sectional study was approved by the Institutional Ethics Committee of Hangzhou Normal University Affiliated Hospital (approval number: 2024E2‐HS‐048) and conducted in accordance with the Declaration of Helsinki. Informed consent was obtained from all participants. Between September 2022 and December 2023, 48 amateur marathon runners from Hangzhou, China, were recruited and stratified into four groups by running experience: Group A (1 year, *n* = 12), Group B (3 years, *n* = 12), Group C (5 years, *n* = 12), and Group D (> 5 years, *n* = 12). A control group of 12 healthy volunteers with no marathon experience and weekly exercise < 150 min was included.

### 2.2 Inclusion and Exclusion Criteria

#### 2.2.1 Inclusion Criteria

1. Amateur marathon enthusiasts with no formal training or profession in marathon running.
2. Age 30–50 years old.
3. Regular running practice with monthly running distance of 100–200 km.
4. Ability to cooperate in completing questionnaires, clinical assessments, and MRI scans.

#### 2.2.2 Exclusion Criteria

1. Contraindications for MRI examination.
2. MRI images indicating partial or complete Achilles tendon rupture.
3. Having a history of Achilles tendon pain in the past year, experiencing pain during rest or exercise, or changing exercise habits due to Achilles tendon discomfort.
4. History of ankle trauma, surgery, or infection.
5. Systemic metabolic diseases (e.g., diabetes and hyperlipidemia), chronic illnesses, or other underlying conditions.
6. Body mass index (BMI) < 18.5 kg/m^2^ or BMI ≥ 28 kg/m^2^.

#### 2.2.3 MRI Data Acquisition

All subjects underwent bilateral ankle MRI scans. Strenuous exercise was avoided 1 day prior to scanning, and a 30‐min rest period was observed before scanning to minimize the influence of prior exercise on the Achilles tendon. The MRI examinations were performed using a GE Discovery MR 750 3.0 T scanner with a dedicated 16‐channel ankle coil. The subjects lie supine with their feet extended forward. The ankle joint is positioned neutrally, at approximately 90° relative to the tibia, whereas the surface of the sole of the foot remains perpendicular to the examination table. The scanning sequences included axial, coronal, and sagittal fat‐suppressed proton density‐weighted imaging (fs‐PDWI) sequences, sagittal T1‐weighted imaging (T1WI) sequences, and sagittal three‐dimensional isotropic fast spin‐echo proton density‐weighted sequences (3D‐CUBE‐PD). The specific scanning sequence parameters are detailed in Table 1.

**TABLE 1:** Scanning sequence parameters.

Sequence	TR (ms)	TE (ms)	Slice thickness (mm)	Slice gap (mm)	FOV (mm × mm)	Flip angle (°)
Axial fs‐PDWI	3312.0	30.0	3.5	1.0	120 × 120	111
Coronal fs‐PDWI	2129	30	3.5	1.0	160 × 160	111
Sagittal fs‐PDWI	2649	41	3.0	0.3	140 × 140	111
Sagittal T1WI	479.0	41	3.0	0.3	140 × 140	111
Sagittal 3D‐CUBE‐PD	2500.0	35.0	2.0	/	180 × 180	Echo train length: 50

*Note:* “/” indicates no slice gap (contiguous scanning). The sagittal 3D‐CUBE‐PD sequence uses an echo train length of 50, and other sequences adopt standard parameters for musculoskeletal MRI to optimize tendon and soft tissue visualization.

Abbreviations: FOV = field of view, TE = echo time, and TR = repetition time.

#### 2.2.4 Image Analysis and Quantitative Measurements

**FIGURE 1:**
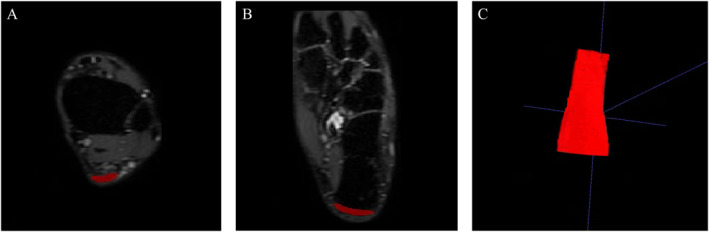
Schematic diagram of MRI for Achilles tendon volume delineation. (A) Delineation diagram of the starting end of the Achilles tendon cross‐section. (B) Delineation diagram of the terminal end of the Achilles tendon cross‐section. (C) Three‐dimensional image of the Achilles tendon obtained by fusion.

Qualitative analysis was performed by two musculoskeletal radiologists (5 years of experience) to identify MRI signs of midportion tendinopathy, insertional tendinopathy, retrocalcaneal bursitis, and paratendinopathy, with discrepancies resolved by a senior radiologist (> 10 years of experience). For quantitative measurements, 3D‐CUBE‐PD images were imported into ITK‐SNAP software (v3.8.0) for multiplanar reconstruction. Tendon length was measured from the soleus muscle‐tendon junction to calcaneal insertion [14]. Tendon thickness was assessed as the anteroposterior diameter at the mid‐tendon on axial images. Tendon volume was calculated via manual segmentation of the tendon from the soleus muscle‐tendon junction to calcaneal insertion, with tendon CSA derived as volume/length [14] (Figure 1). Interobserver reliability was evaluated using intraclass correlation coefficients (ICC).

The qualitative diagnosis of the Achilles tendon included the MRI manifestations of midportion tendinopathy, insertional tendinopathy, retrocalcaneal bursitis, superficial calcaneal bursitis, and paratendinopathy [15]. The anatomical locations and MRI manifestations of the corresponding diseases are detailed in Table 2.

**TABLE 2:** Anatomical locations and MRI findings of Achilles tendon‐related disorders.

Achilles tendon‐related disorders	Anatomical location	MRI findings
Midportion Achilles tendinopathy	2–7 cm from the calcaneal insertion site	fs‐T1WI or T2WI: Fusiform expansion with central hyperintensity consistent with intratendinous neovascularization
Insertional Achilles tendinopathy	Insertion zone of the Achilles tendon on the calcaneus (frequently associated with spur formation and calcification at the insertion site)	fs‐T1WI or T2WI: Osteophyte formation and/or hyperintensity at the tendon insertion site
Retrocalcaneal bursitis	Bursa between the anterior‐inferior Achilles tendon and posterior‐superior calcaneus	T2WI: Retrocalcaneal bursal effusion exceeding normal physiological volume (anteroposterior × superoinferior × transverse diameter: > 1 × 7 × 11 mm)
Superficial calcaneal bursitis	Bursa between the Achilles tendon and overlying skin	T2WI: Hyperintensity between the tendon and subcutaneous tissue
Paratendinopathy	Peritendinous membrane surrounding the Achilles tendon	fs‐T1WI or T2WI: Peritendinous hyperintensity

*Note:* Size criteria for retrocalcaneal bursal effusion are provided for clinical reference. MRI findings reflect pathological changes such as tendon degeneration, neovascularization, bursal inflammation, and peritendinous edema, with fat‐saturated sequences enhancing tissue contrast for improved lesion detection.

Abbreviations: fs‐T1WI = fat‐saturated T1‐weighted imaging, T2WI = T2‐weighted imaging.

#### 2.2.5 Statistical Analysis

Data were analyzed using SPSS 23.0. Normality was tested via the Shapiro–Wilk test, with parametric data reported as mean ± SD and nonparametric data as median (IQR). Group differences in demographic characteristics and MRI metrics were assessed using one‐way ANOVA (parametric) or Kruskal–Wallis H test (nonparametric), with post hoc Bonferroni correction for pairwise comparisons. Chi‐squared tests evaluated between‐group differences in pathology prevalence. ICC values > 0.75 indicated excellent interobserver agreement. A significance threshold of *p* < 0.05 was applied throughout.

## 3 Results

### 3.1 Baseline Characteristics

To evaluate the comparability of demographic and running‐related parameters across different running experience groups and the control group.

A total of 60 amateur marathon runners (12 in each group: A [1 year], B [3 years], C [5 years], and D [> 5 years]) and 12 healthy controls underwent bilateral ankle MRI. Demographic data showed no significant differences in sex, age, height, weight, BMI, or monthly running distance among groups (all > 0.05) (Table 3). All groups were comparable in key baseline characteristics.

**TABLE 3:** Comparison of baseline data.

Baseline characteristics	Control group (*n* = 12)	Group A (*n* = 12)	Group B (*n* = 12)	Group C (*n* = 12)	Group D (*n* = 12)	Statistic	*p* value
Gender						4.20	0.379
Male	7 (58.3)	9 (75.0)	9 (75.0)	10 (83.3)	11 (91.7)		
Female	5 (41.7)	3 (25.0)	3 (25.0)	2 (16.7)	1 (8.3)		
Age (years)	38.7 ± 3.9 (32∼44)	33.0 (30.5,34.0) (30∼46)	37.9 ± 5.0 (31∼46)	40.2 ± 4.7 (30∼45)	42.0 (39.0,43.5) (30∼50)	7.87	0.097
Height (m)	1.68 ± 0.09 (1.55∼1.82)	1.69 ± 0.06 (1.58∼1.77)	1.70 ± 0.06 (1.60∼1.78)	1.69 ± 0.06 (1.60∼1.80)	1.70 ± 0.05 (1.63∼1.78)	0.27	0.895
Weight (kg)	59.50 ± 9.14 (50∼78)	61.58 ± 7.56 (47∼75)	65.00 ± 8.49 (48∼77)	65.75 ± 8.64 (51∼80)	63.75 ± 5.75 (54∼74)	1.23	0.308
BMI (kg/m^2^)	21.06 ± 1.30 (19.49∼23.55)	21.61 ± 1.43 (18.36∼23.94)	22.53 ± 1.69 (18.29∼24.31)	22.53 ± 1.32 (19.92∼24.69)	22.00 ± 1.57 (19.13∼24.44)	2.19	0.082
Monthly running distance (km)	—	120 (120,150) (100∼160)	200 (110, 200) (100∼200)	190 (150,200) (100∼200)	135 (100,175) (100∼200)	7.11	0.068

*Note:* Data are presented as *n* (%), mean ± SD (range), or median (IQR) (range). The control group had no reported monthly mileage as they were nonrunners. Statistic values correspond to chi‐squared tests for categorical variables (gender) or Kruskal–Wallis tests for continuous variables (age, height, weight, BMI, and monthly running distance). All baseline characteristics showed no statistically significant differences across groups (*p* > 0.05).

Abbreviation: BMI = body mass index.

### 3.2 Interobserver Reliability

To assess the reproducibility of quantitative Achilles tendon measurements between two observers.

Interobserver agreement for tendon length, thickness, volume, and mean CSA was evaluated using intraclass correlation coefficients (ICC), which all exceeded 0.75, indicating excellent consistency. The ICC for Achilles tendon length was 0.932, accompanied by a 95% confidence interval ranging from 0.901 to 0.954. For Achilles tendon thickness, the ICC was 0.901, with a 95% confidence interval of 0.789–0.922. The ICC for Achilles tendon volume was 0.857, with a 95% confidence interval of 0.738–0.932. Finally, the ICC for CSA was 0.872, with a 95% confidence interval spanning from 0.699 to 0.912.

The ICC values for all parameters exceeded 0.75, demonstrating excellent reliability between the two observers in assessing each Achilles tendon measurement parameter. This confirms the robustness of the quantitative measurement approach and validates the morphological data obtained from the images.

### 3.3 Qualitative MRI Findings

**FIGURE 2:**
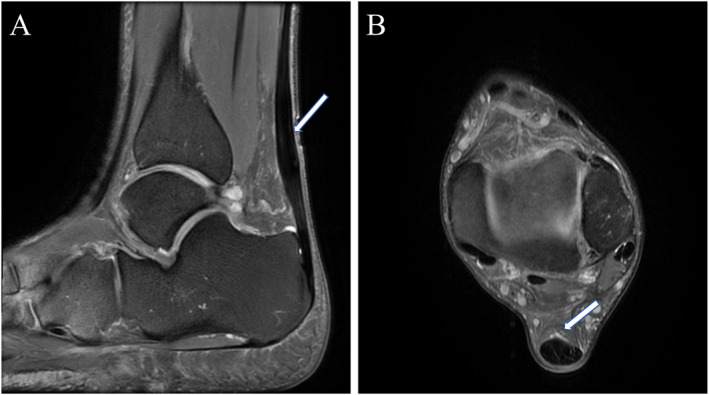
A 50‐year‐old male amateur marathon runner with a 7‐year running experience and a monthly running distance of 200 km; representative MRI of midsection Achilles tendinopathy. (A) Sagittal fs‐PDWI of the left ankle shows fusiform expansion of the mid‐Achilles tendon with linear hyperintense signals (arrow) and (B) axial fs‐PDWI of the left ankle demonstrates thickening of the Achilles tendon, increased anteroposterior diameter, and heightened internal signal (arrow).

**FIGURE 3:**
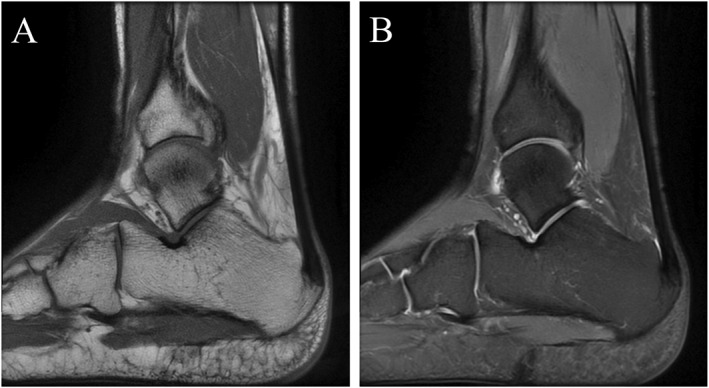
A 32‐year‐old male amateur marathon runner with a 5‐year running experience and a monthly running distance of 150 km; representative MRI of inserted Achilles tendinopathy. Sagittal T1WI (A) and fs‐PDWI (B) of the right ankle show calcaneal spur formation and linear hyperintense signal enhancement at the Achilles tendon insertion site.

**FIGURE 4:**
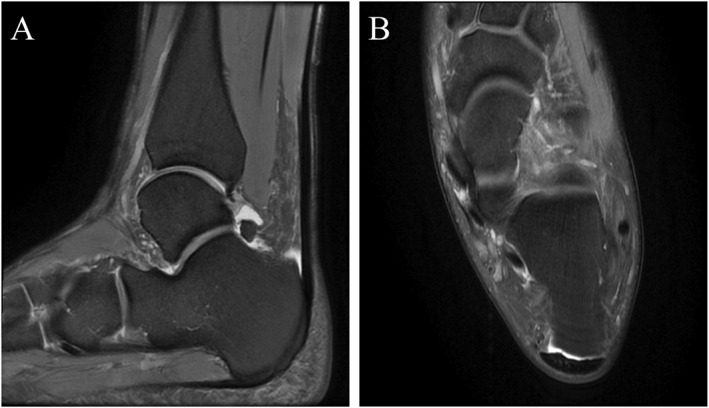
A 31‐year‐old male amateur marathon runner with a 3‐year running experience and a monthly running distance of 120 km; representative MRI of posterior bursitis. Sagittal (A) and axial fs‐PDWI (B) of the left ankle show increased retrocalcaneal bursal effusion (anteroposterior diameter × superoinferior diameter × transverse diameter: 5 × 9 × 12 mm).

**FIGURE 5:**
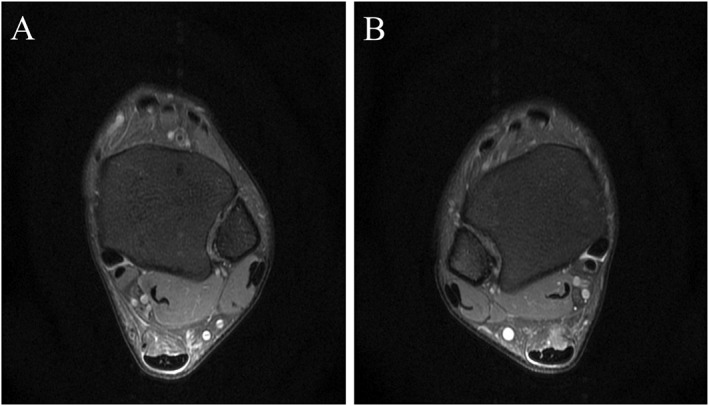
A 37‐year‐old male amateur marathon runner with a 3‐year running experience and a monthly running distance of 200 km; representative MRI images of paratendinopathy. (A–B) Axial fs‐PDWI of bilateral ankles shows circumferential hyperintense signal enhancement around the Achilles tendon.

To determine the prevalence of asymptomatic Achilles tendon pathologies and their association with running experience. The MRI manifestations revealed that among 96 sides of Achilles tendons from amateur marathon runners, there were 20 cases (20/96, 20.83%) of midportion Achilles tendinopathy (Figure 2), 24 cases (24/96, 25.00%) of insertional Achilles tendinopathy (Figure 3), 39 cases (39/96, 40.63%) of retrocalcaneal bursitis (Figure 4), and 4 cases (4/96, 4.16%) of Achilles paratendinopathy (Figure 5). In Groups A, B, C, and D (24 sides each), the incidences of midportion tendinopathy were 2 (8.33%), 3 (12.50%), 7 (29.17%), and 8 (33.33%), insertional tendinopathy were 3 (12.50%), 4 (16.67%), 8 (33.33%), and 9 (37.50%), and retrocalcaneal bursitis were 2 (8.33%), 9 (37.50%), 13 (54.17%), and 15 (62.50%), respectively, with additional 2 cases (8.33%) of paratendinopathy in Groups B and D. The control group (12 subjects and 24 sides) had 2 cases (8.33%) each of insertional tendinopathy and retrocalcaneal bursitis. Superficial calcaneal bursitis was not observed in all subjects. Statistically significant differences were observed in the incidence rates of midportion Achilles tendinopathy, insertional Achilles tendinopathy, and retrocalcaneal bursitis between the control group and amateur marathon runner groups with different running ages (Table 4).

**TABLE 4:** Results of Achilles tendon MRI findings.

MRI findings	Control group (*n* = 12)	Group A (*n* = 12)	Group B (*n* = 12)	Group C (*n* = 12)	Group D (*n* = 12)	Statistic	*p* value
Midportion Achilles tendinopathy	0 (0)	2 (8.33)	3 (12.50)	7 (29.17)	8 (33.33)	16.75	0.002
Insertional Achilles tendinopathy	2 (8.33)	3 (12.50)	4 (16.67)	8 (33.33)	9 (37.50)	9.65	0.047
Retrocalcaneal bursitis	2 (8.33)	2 (8.33)	9 (37.50)	13 (54.17)	15 (62.50)	24.76	< 0.001
Paratendinopathy	0 (0)	0 (0)	2 (8.33)	0 (0)	2 (8.33)	—	—
Superficial calcaneal bursitis	0 (0)	0 (0)	0 (0)	0 (0)	0 (0)	—	—

*Note:* Data are presented as *n* (%). Statistic values correspond to chi‐squared tests for group comparisons. Significant differences were observed in the incidence of midportion Achilles tendinopathy (*p* = 0.002), insertional Achilles tendinopathy (*p* = 0.047), and retrocalcaneal bursitis (*p* < 0.001) across groups, with higher frequencies in amateur marathon runner groups (A–D) compared to the control group. Paratendinopathy showed low prevalence and no significant group‐wise association due to limited cases (*p* > 0.05). Results suggest a potential correlation between running experience and the development of Achilles tendon‐related pathologies.

The incidence of asymptomatic tendon pathologies increased significantly with longer running experience, highlighting running experience as a risk factor for subclinical tendon changes.

### 3.4 Quantitative Morphological Changes

To investigate the impact of running experience on quantitative morphological parameters of the Achilles tendon. Tendon length showed no significant group differences (*p* = 0.433). Tendon thickness, volume, and mean CSA were significantly higher in long‐experience running groups (C and D) compared to short‐experience groups (A and B) and the control group. Specifically, Group A had lower thickness than Groups C and D (*p* = 0.021 and *p* = 0.036), whereas Groups C and D exhibited larger volume and CSA than the control group (*p* ≤ 0.043) and Group A (*p* ≤ 0.004; Table 5).

**TABLE 5:** Comparison of Achilles tendon morphological quantitative values among.

MRI morphological indexes of Achilles tendon	Control group (*n* = 12)	Group A (*n* = 12)	Group B (*n* = 12)	Group C (*n* = 12)	Group D (*n* = 12)	Statistic	*p* value
Length (mm)	61.22 ± 5.97	63.08 ± 9.42	64.69 ± 9.14	63.50 (56.64, 68.68)	63.59 (60.22, 68.84)	3.80	0.433
Thickness (mm)	5.48 ± 0.67	5.05 ± 0.73	5.29 (5.04, 5.71)	5.73 (5.07, 6.81)	5.56 (5.25, 5.98)	12.50	0.014
Volume (mm^3^)	2636.04 (2323.21,2832.87)	2617.65 (2343.86, 3244.02)	2996.36 ± 671.82	3468.19 (2643.41, 4143.89)	3600.05 ± 700.86	22.48	< 0.001
Mean CSA (mm^2^)	42.23 (39.44,47.29)	43.74 ± 8.27	46.03 ± 6.34	49.20 (43.63, 65.50)	53.65 ± 6.69	30.88	< 0.001

*Note:* Data are presented as mean ± SD, median (IQR), or median (range) based on normality. Statistic values correspond to Kruskal–Wallis tests for non‐normally distributed data or one‐way ANOVA for normally distributed data. Significant group differences were observed in thickness (*p* = 0.014), volume (*p* < 0.001), and mean CSA (*p* < 0.001), with progressive increases in morphological indices from the control group to amateur marathon runner groups (A–D). No significant difference was found in tendon length (*p* > 0.05). The results indicate that long‐term running may be associated with structural adaptations (e.g., thickening and increased volume) of the Achilles tendon, potentially reflecting degenerative changes or compensatory hypertrophy.

Abbreviation: CSA = cross‐sectional area.

Prolonged running experience is associated with morphological remodeling of the Achilles tendon, manifested as increased thickness, volume, and CSA, without significant changes in tendon length.

## 4 Discussion

Our study found a positive link between running experience and increased Achilles tendon thickness, CSA, and volume across different experience levels, likely due to structural adaptations to cumulative loads. However, the cross‐sectional design and potential confounding factors, such as variations in monthly mileage and the mix of asymptomatic and pathological cases within groups, suggest that these results should be viewed as trends rather than conclusive evidence of causation.

Runners with ≥ 5 years of running experience showed significantly higher prevalence of retrocalcaneal bursitis (62.50%), midportion tendinopathy (33.33%), and, insertional tendinopathy (37.50%) compared to short‐experience groups (≤ 3 years) and controls, aligning with prior findings that cumulative mechanical loading drives progressive microdamage. The retrocalcaneal bursa serves as a cushioning mechanism during marathons by alleviating local pressure and friction. However, as amateur marathon runners accumulate experience, they may encounter a sustained increase in pressure within the retrocalcaneal bursa. This heightened pressure often results from excessive exercise coupled with inadequate recovery, ultimately leading to the development of retrocalcaneal bursitis [15]. Increased pressure applies mechanical stress to the Achilles tendon, disrupting the orderly arrangement of its fibers. This disruption, coupled with inflammation, accelerates collagen degradation and results in structural changes within the tendon, ultimately manifesting as swelling and pain [16, 17]. In recent years, the incidence of Achilles tendinopathy has risen, making it the most common pathological condition among running‐related injuries [18], with reported incidence rates ranging from 5% to 20% [19, 20]. Among amateur marathon runners, the incidences of both insertional and midcourse Achilles tendinopathy are notably elevated, progressively increasing across various age groups of runners. This trend may be attributed to degeneration of the Achilles tendon caused by the repetitive and excessive stresses associated with marathon running, which complicates complete recovery. Lieberthal et al. [13] reported a high incidence of Achilles tendon lesions among asymptomatic male long‐distance runners, observing that this prevalence correlates with an increase in running age. These findings are consistent with the results of the current study.

Research indicates that approximately 34.2% of asymptomatic marathon runners display hypoechoic Achilles tendons, suggesting potential ultrasound evidence of Achilles tendinopathy, a result that aligns closely with our own findings [21]. However, our study enhances these results by establishing a clear dose‐response relationship with running experience. Specifically, each additional year of running increased the odds of developing tendinopathy, a trend not explicitly noted in previous cross‐sectional studies. This observation supports the hypothesis that running experience serves as a cumulative risk factor, with prolonged exposure exceeding the tendon's repair capacity.

Long‐experience runners also exhibited 15%–20% greater tendon thickness, volume, and CSA than novices, reflecting adaptive hypertrophy in response to repetitive stress, a trend consistent with ultramarathon studies showing tendon diameter increases after extreme distances [22]. Achilles tendon thickening correlates with an increase in the weekly training distance among adult competitive runners and varies throughout the training cycle. Devaprakash et al. [14] reported that trained middle and long‐distance runners exhibited larger CSA compared to a control group, a difference attributed to collagen fiber recombination and tendon cell proliferation. Furthermore, the thickness of the Achilles tendon is linked to improved long‐distance running performance as it augments the ability to absorb and transmit ground reaction forces [23, 24, 25]. The observed morphological alterations likely reflect a dynamic balance between adaptive and degenerative processes: increased CSA and thickness in long‐experience runners represent a compensatory response to chronic mechanical stress, with studies showing repetitive loading induces collagen synthesis to enhance tendon stiffness, though this adaptation may transition to degenerative changes, such as collagen disorganization, when mechanical demands exceed repair capacity [26]. This indicates that an increase in the CSA and thickening of the Achilles tendon are physiological adaptations to repetitive loads, potentially benefiting runners in training and competition. Nevertheless, these adaptations may also be associated with the onset of Achilles tendinopathy and the risk of future injuries.

Moreover, the study findings suggest that the tendon lengths in all groups remained constant, indicating that remodeling primarily entails collagen reorganization and fiber alignment within the tissue rather than structural elongation. This aligns with previous research on tendon adaptation to mechanical stress [27, 28]. It is worth noting that most researchers believe that changes in the tendon lengths have little impact on the risk of injury [29]. Such findings are consistent with existing research on tendon adaptation to mechanical loads. In our cohort, a 15%–20% increase in CSA among runners with a duration of ≥ 5 years may represent the compensatory stage, whereas the higher prevalence of tendon lesions in this group (33.33% of midrange tendon lesions) indicates the coexistence of cumulative microinjuries and adaptability. The observed morphological changes may reflect a dynamic balance between adaptive and degenerative processes: the increase in CSA and thickness in experienced runners represents a compensatory response to chronic mechanical stress, with repeated loads inducing collagen synthesis to enhance tendon stiffness. Research indicates that tendons exhibiting a CSA increase of over 50% are more susceptible to clinical symptoms. This finding suggests that exceeding a certain threshold may hinder structural adaptation's ability to meet mechanical demands [30]. Consequently, there appears to be a threshold beyond which adaptation gives way to degeneration. This concept will be a central focus of our future research.

It is important to highlight that in this study, asymptomatic athletes were not categorized directly by monthly running volume. Nevertheless, initial correlation analysis revealed a positive correlation between monthly running distance and the CSA of the Achilles tendon (*r* = 0.538 and *p* = 0.010). This implies that running distance could significantly influence the Achilles tendon's adaptability. In future research, we plan to integrate running distance as a key variable in the statistical model to differentiate the distinct impacts of “ running experience” and “running distance.”

The study provides imaging evidence regarding managing Achilles tendon health in amateur marathon runners. Runners with over 5 years of experience show increased Achilles tendon thickness, volume, and cross‐sectional area, correlating with higher incidences of tendinitis and bursitis. This suggests a link between adaptive hypertrophy and subclinical degeneration due to prolonged running. Even asymptomatic runners are at risk for future symptoms, emphasizing the importance of recognizing associated risks. Screening for abnormal Achilles tendon structure is recommended for runners with 3–5 years of consistent long‐distance running, particularly before increasing training or competing. It is crucial to consider years of running experience when assessing Achilles tendon injury risk. Experienced runners, with over 5 years of running, should receive guidance on tendon protection and undergo regular imaging monitoring, regardless of symptoms.

This study has several notable limitations that warrant consideration. First, the absence of histopathological confirmation for MRI‐detected tendon abnormalities represents a critical gap as qualitative interpretations of tendinopathy or bursitis rely solely on imaging features without histological gold standard validation. Second, the cross‐sectional design precludes causal inference, limiting the ability to determine whether observed morphological changes precede or follow the development of subclinical pathologies. The relatively small sample size (12 participants per group), whereas typical for MRI‐based musculoskeletal studies, may reduce the power to detect subtle group differences, particularly for rare findings such as paratendinopathy. Furthermore, the study population was restricted to asymptomatic amateur runners in a single geographic region, potentially limiting the generalizability of the findings to professional athletes or to populations with different training regimens. Additionally, recruitment for this study was not gender‐stratified, resulting in a cohort that included only 18.6% females. Therefore, caution is warranted when extrapolating these findings to female runners. Lastly, potential modifiers of tendon health—such as specific training intensity, footwear type, footstrike pattern, or prior lower limb injuries—were not systematically assessed, introducing unmeasured confounding variables. Future longitudinal studies with larger cohorts, histopathological correlation, and multivariate adjustment for systemic and behavioral factors are needed to validate these findings and clarify the temporal relationship between running experience, morphological remodeling, and clinical outcomes.

## 5 Conclusion

This cross‐sectional study used MRI to analyze Achilles tendon morphology and pathological changes in 60 asymptomatic amateur marathon runners categorized by running experience (1, 3, 5, and > 5 years) and 12 nonrunning controls. Key findings show that longer running experience correlates with increased prevalence of asymptomatic pathologies: midportion tendinopathy (0%–33.3%), insertional tendinopathy (8.3%–37.5%), and retrocalcaneal bursitis (8.3%–62.5%), with all *p* < 0.05. Quantitatively, tendons in long‐experience groups (C/D, ≥ 5 years) exhibited significantly greater thickness, volume, and mean CSA than short‐experience groups (A/B) and controls (all *p* < 0.05), while tendon length showed no significant differences. Interobserver reliability for MRI measurements was excellent (ICC > 0.75). These results indicate that prolonged running induces morphological remodeling (hypertrophy) and subclinical degenerative changes in the Achilles tendon. MRI effectively detects early asymptomatic pathologies, supporting its use for monitoring high‐risk runners to facilitate timely injury prevention. Running experience exceeding 5 years is associated with an increased likelihood of morphological changes in the Achilles tendon and the emergence of subclinical lesions. Consequently, clinicians should conduct regular imaging screenings for amateur runners with significant running experience. They should assess the risks based on the duration of the runners' practice and develop tailored preventive strategies to delay the onset of symptomatic injuries.

## Author Contributions

**Wanzhen Yao:** conceptualization, data curation, formal analysis, writing – original draft, writing – review and editing. **Yong Chen:** conceptualization, writing – original draft, writing – review and editing. **Siyu Dai:** conceptualization, writing – review and editing. **Jing Zhou:** writing – review and editing, data curation. **Xinmiao Mao:** writing – review and editing. **Yanjing Zhang:** writing – review and editing, data curation. **Jianping Ding:** writing – original draft, writing – review and editing. **Jie Liu:** supervision, writing – review and editing, visualization. **Jie Huang:** funding acquisition, project administration, writing – review and editing.

## Funding

The study was partly funded by Hangzhou Medical and Health Science and Technology Project (Grants A20220387, ZD20240006, and A20251077), Hangzhou Biomedicine and Health Industry Development Supporting Science and Technology Special Project (Grant 2024WJC012), Zhejiang Province Medical and Health Science and Technology Program (Grant 2022KY260), and the key medical Disciplines of Hangzhou (Grant YDYX).

## Ethics Statement

Informed consent was obtained from all persons before their participation.

## Consent

The authors have nothing to report.

## Conflicts of Interest

The authors declare no conflicts of interest.

## Data Availability

The datasets used and/or analyzed for this study are available from the corresponding author upon reasonable request.
